# Long-Distance Translocation of Protein during Morphogenesis of the Fruiting Body in the Filamentous Fungus, *Agaricus bisporus*

**DOI:** 10.1371/journal.pone.0028412

**Published:** 2011-12-06

**Authors:** Benjamin M. Woolston, Carl Schlagnhaufer, Jack Wilkinson, Jeffrey Larsen, Zhixin Shi, Kimberly M. Mayer, Donald S. Walters, Wayne R. Curtis, C. Peter Romaine

**Affiliations:** 1 Department of Chemical Engineering, Pennsylvania State University, University Park, Pennsylvania, United States of America; 2 Department of Plant Pathology, Pennsylvania State University, University Park, United States of America; 3 Agarigen, Inc., Durham, North Carolina, United States of America; Universidade de Sao Paulo, Brazil

## Abstract

Commercial cultivation of the mushroom fungus, *Agaricus bisporus*, utilizes a substrate consisting of a lower layer of compost and upper layer of peat. Typically, the two layers are seeded with individual mycelial inoculants representing a single genotype of *A. bisporus*. Studies aimed at examining the potential of this fungal species as a heterologous protein expression system have revealed unexpected contributions of the mycelial inoculants in the morphogenesis of the fruiting body. These contributions were elucidated using a dual-inoculant method whereby the two layers were differientially inoculated with transgenic β-glucuronidase (GUS) and wild-type (WT) lines. Surprisingly, use of a transgenic GUS line in the lower substrate and a WT line in the upper substrate yielded fruiting bodies expressing GUS activity while lacking the *GUS* transgene. Results of PCR and RT-PCR analyses for the *GUS* transgene and RNA transcript, respectively, suggested translocation of the GUS protein from the transgenic mycelium colonizing the lower layer into the fruiting body that developed exclusively from WT mycelium colonizing the upper layer. Effective translocation of the GUS protein depended on the use of a transgenic line in the lower layer in which the *GUS* gene was controlled by a vegetative mycelium-active promoter (laccase 2 and β-actin), rather than a fruiting body-active promoter (hydrophobin A). GUS-expressing fruiting bodies lacking the *GUS* gene had a bonafide WT genotype, confirmed by the absence of stably inherited GUS and hygromycin phosphotransferase selectable marker activities in their derived basidiospores and mycelial tissue cultures. Differientially inoculating the two substrate layers with individual lines carrying the *GUS* gene controlled by different tissue-preferred promoters resulted in up to a ∼3.5-fold increase in GUS activity over that obtained with a single inoculant. Our findings support the existence of a previously undescribed phenomenon of long-distance protein translocation in *A. bisporus* that has potential application in recombinant protein expression and biotechnological approaches for crop improvement.

## Introduction

The mushroom-bearing fungus, *Agaricus bisporus*, is a common soil inhabitant that thrives through the secondary decomposition of dead and decaying plant material associated with forests and grasslands in North America and Europe [1], [2]. Apart from playing a role as a decomposer of plant litter in its ecological niche, the commercial cultivation of this fungal species, commonly known as white button, portabella, and crimini mushrooms, represents a major agricultural enterprise worldwide [3].

*Agaricus bisporus* is commercially cultivated in a composted mixture of plant and animal waste products [4]. The prepared compost is seeded with sterilized cereal grain colonized by the vegetative mycelium of *A. bisporus*. Once colonized by the mushroom fungus, the compost is overlaid with a layer of peat that serves as a water reservoir for the developing fruiting body (mushroom). A common practice is to seed the upper peat layer with a second mycelial inoculant [5], [6]. Cellular fusion of the mycelia occurring at the junction of the upper and lower substrates results in the formation of a singular mycelial network, thereby allowing for the symplastic movement of nutrients from the compost-borne mycelium into the developing fruiting body. Within three weeks of applying the peat layer, fruiting bodies form and continue to develop in a rhythmic fashion at about weekly intervals.

Since the commercial cultivation process provides for the large-scale, rapid, and inexpensive production of biomass, and methods are available for transgenic manipulation, studies were undertaken to examine *A. bisporus* as an expression system for recombinant proteins, such as biologics and industrial enzymes. Herein, we report on the expression patterns of the β-glucuronidase (GUS) reporter protein in the fruiting body when transgenic lines carrying different *GUS* gene promoters were employed as inoculants for the growth substrate. We provide evidence that the genotype of the fruiting body is determined largely by the genotype of the *A. bisporus* inoculant used in the upper substrate layer and demonstrate the long-distance translocation of GUS protein from the compost-borne mycelium into the developing fruiting body.

## Results and Discussion

### Dual-inoculant method allows a determination of the relative contribution of substrate inoculants

**Figure 1:**
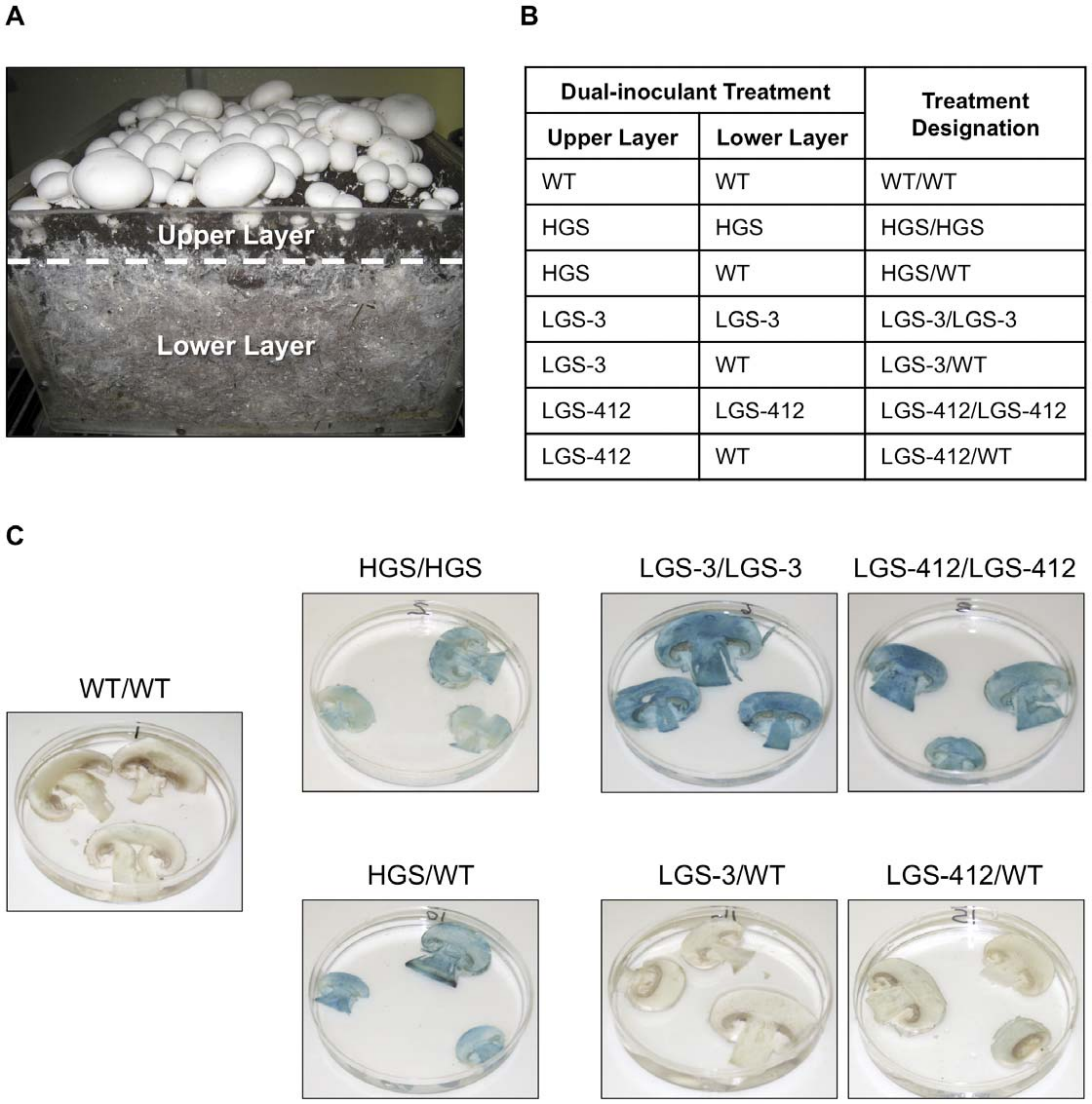
Dual-inoculant method. (A) Cross-section of the bi-layered cultivation substrate for *Agaricus bisporus* showing the lower bed of compost (Lower Layer) and peat overlay (Upper Layer). Cellular fusion of the mycelia growing in the peat and compost occurs at the interface of the two layers (dashed line), forming a singular network throughout the cultivation substrate. (B) Notation system for dual-inoculant treatments shown in Fig. 1C. (C) GUS activity in fruiting bodies grown using wild-type and transgenic inoculants. WT: wild-type line; GUS lines carrying the hydrophobin A promoter (HGS) and laccase 2 promoter (LGS); 3 and 412 denote independent transformants.

Our experimental protocol for producing fruiting bodies of *A. bisporus* employed the conventional bi-layered substrate consisting of an upper peat layer and a lower compost layer (Fig. 1A). To examine protein expression patterns, we used a dual-inoculant method whereby the two layers were differentially inoculated with a WT line and a transgenic line carrying the *GUS* gene controlled by a native promoter. Most studies employed line HGS carrying the fruiting body-specific hydrophobin A (*HYPHA*) promoter [7], line LGS carrying the vegetative mycelium-active laccase 2 (*LCC2*) promoter [8], [9], and line AGS with the constitutive β-actin (*ACTN*) promoter [10], [11]. Details for the various transgenic lines, promoters, and transgenic notations are provided in Table 1. By using two different inoculants, the level of GUS enzyme activity in the fruiting body provided a measure of the individual roles of the vegetative mycelia colonizing the upper and lower substrates. Our notation system for a dual-inoculant treatment was expressed as a fraction, where the numerator and denominator indicated the upper- and lower-layer inoculants, respectively (Fig. 1B).

**Table 1:** Summary of the GUS lines used in the present study.

Line	*Agaricus* Gene^a^	Protein ID	Fruiting Body Rank^b^	VegetativeMycelium Rank^b^	Fruiting Body: Mycelium Ratio^c^
HGS	Hydrophobin A (*HYPA*)	133693	1	2,488	108
DGS	Fruiting body-specific D (*FBSD*)	193061	5	1,180	27
LnGS	Lectin (*LCTN*)	194888	11	62	2.4
AGS	β-actin (*ACTN*)	192120	86	79	0.975
LGS	Laccase 2 (*LCC2*)	135709	7,127	114	0.007

a Promoter used to drive expression of the *GUS* gene.

b Transcriptomic data represent the expression of each gene relative to the other 10,413 genes in the *A. bisporus* genome.

c The ratio is calculated from the raw expression data and not from the rank.

Throughout our study, two or three independent transformants for each of the lines HGS, LGS, and AGS were employed for the dual-inoculant method. Independent transformants for a particular GUS line varied in their level of GUS activity, but displayed an identical behavior as an inoculant. In all cases, the use of a GUS line either alone or paired with a WT line as an inoculant treatment resulted in a normal timing of development and morphology of the fruiting body.

### Fruiting body genotype is determined by the upper-layer inoculant

Initial experiments compared the level of GUS enzyme activity in fruiting bodies grown using either a transgenic inoculant in the upper layer and a WT inoculant in the lower layer or a transgenic inoculant in both layers. We observed that the promoter used to express the *GUS* gene had a profound effect on the behavior of the transgenic line when used as an inoculant. For example, when line HGS carrying the *GUS* gene controlled by the *HYPA* promoter was used to inoculate the upper layer and with a WT line-inoculated lower layer (HGS/WT), the fruiting bodies expressed 80% of the GUS activity observed for the treatment HGS/HGS where both layers were seeded with the transgenic inoculant (Fig. 1C **;** Table 2). In sharp contrast, employing either of two independent transformants (3 and 412) of line LGS that carried the *GUS* gene driven by the *LCC2* promoter resulted in a<5% retention of GUS activity (LGS-3/WT and LGS-412/WT).

**Table 2:** Quantitative GUS activity assay of fruiting bodies grown using wild-type and transgenic inoculants.

Agaricus Line^a^	GUS Activity^b^	
	Single Inoculant^c^	Dual Inoculant^d^
WT	0.07^e^ (0.03–0.10)	-
HGS	1.04 (1.02–1.05)	0.83 (0.78–0.88)
LGS-3	19.14 (18.2–20.1)	0.26 (0.26–0.26)
LGS-412	5.30 (4.9–5.8)	0.13 (0.12–0.15)

a WT: wild-type line; GUS lines carrying the hydrophobin A promoter (HGS) and laccase 2 promoter (LGS); 3 and 412 denote independent transformants.

b nmol MUG hydrolyzed/minute/100 µg total soluble protein.

c Both upper and lower layers were inoculated with the indicated line.

d Upper layer was inoculated with the indicated line and lower layer with a WT line.

e Value represents the mean, where n = 2; (range).

Despite the marked reduction in enzyme activity using line LGS in the upper layer alone, the findings of qPCR analysis revealed a comparable *GUS* transgene dose in LGS/LGS- and LGS/WT-fruiting bodies with high and low GUS activity, respectively (Table 3). Therefore, the dramatic loss of GUS activity observed with line LGS was not attributed to a diminution of the transgene dose, reflecting a chimeric fruiting body formed by the intermixing of WT and transgenic mycelia. Moreover, these findings were consistent with the mycelium in the upper layer playing a dominant role in formation of the fruiting body, as the presence of WT mycelium in the lower layer did not dilute the transgene dose in the fruiting body.

**Table 3:** qPCR analysis of the *GUS* gene in fruiting bodies grown using wild-type and transgenic inoculants.

Upper Layer Inoculant/Lower Layer Inoculant^a^	Relative *GUS* Gene Value^b^
HGS/HGS	571 (±124)
HGS/WT	642 (±129)
LGS-3/LGS-3	2,734 (±712)
LGS-3/WT	2,711 (±330)
LGS-412/LGS-412	517 (±98)
LGS-412/WT	659 (±34)

a WT: wild-type line; GUS lines carrying the hydrophobin A promoter (HGS) and laccase 2 promoter (LGS); 3 and 412 denote independent transformants.

b Value represents the mean normalized to the endogenous β-actin gene, where n = 3; (s.d.).

**Figure 2:**
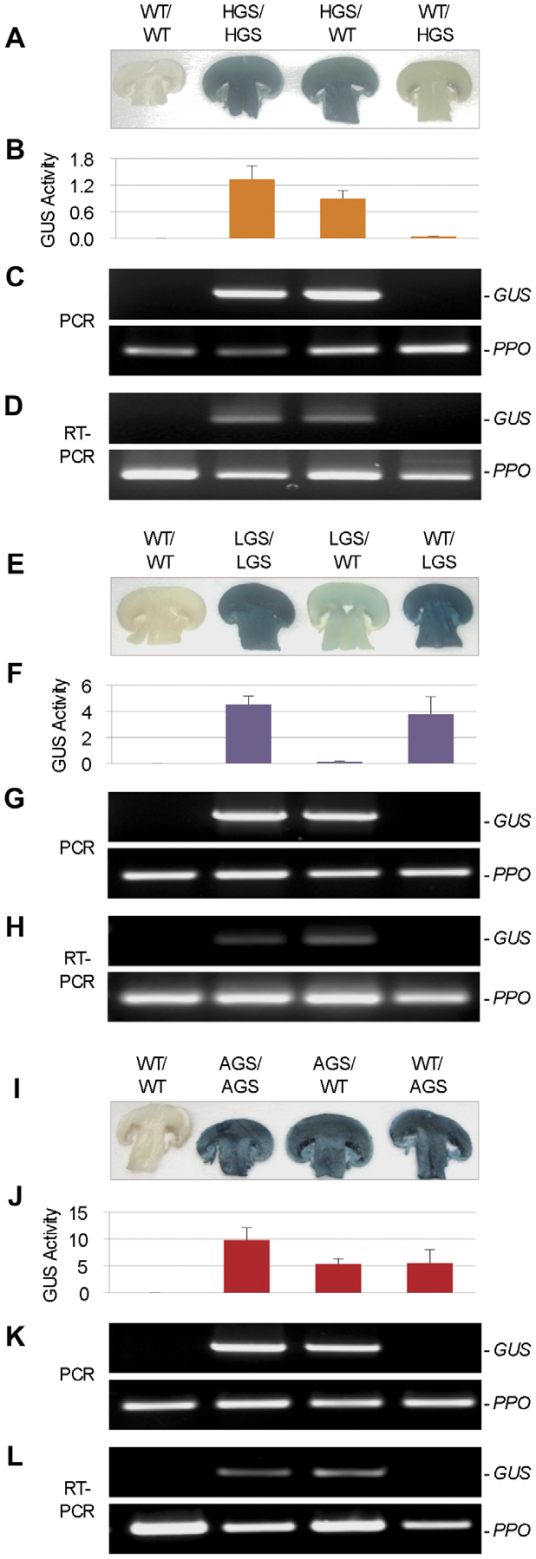
GUS activity assay and molecular analyses of fruiting bodies grown using wild-type and transgenic inoculants. Indicated is the upper layer inoculant/lower layer inoculant for the bi-layered cultivation substrate. WT: wild-type line; GUS lines carrying the hydrophobin A promoter (HGS), laccase 2 promoter (LGS) and β-actin promoter (AGS). (A–D) Line HGS. (E–H) Line LGS. (I–L) Line AGS. (A, E, I) Histological GUS assay. (B, F, J) Quantitative GUS assay. Enzyme activity is expressed as nmol MUG hydrolyzed/minute/100 µg total soluble protein, and represents the mean value of two independent experiments. (C, G, K) PCR analysis of the *GUS* gene. The predicted 163-bp *GUS* amplicon (*GUS*) and 403-bp amplicon for the endogenous polyphenol oxidase 1 (*PPO*) gene, included as a PCR control, are indicated. (D, H, L) RT-PCR analysis of the *GUS* transcript. Indicated are the predicted 163-bp and 403-bp amplicons for the *GUS* transcript and endogenous *PPO* transcript control, respectively.

To further elucidate the relationship between GUS activity and genotype as a function of the spatial positioning of transgenic lines LGS, HGS, and AGS in the growth substrates, fruiting bodies were subjected to PCR and RT-PCR analyses for the *GUS* gene and RNA transcript, respectively. Irrespective of the GUS line and promoter used to express the *GUS* gene, the genotype of the fruiting body was determined by the genotype of the inoculant in the upper layer. Thus, the 163-bp *GUS* amplicon, which was indicative of the *GUS* gene and mRNA, was detected in the fruiting body only when a transgenic inoculant was applied to the upper layer (Fig. 2C, D, G, H, K, L). This finding agreed with the results of qPCR analysis (Table 3) revealing that the transgene dose was unaffected by a WT-colonized lower layer. Hence, the upper-layer mycelium appears to play a primary role in the formation of the primordium giving rise to the fruiting body, quite possibly to the near-complete exclusion of the mycelium colonizing the lower layer. However, the PCR and qPCR data could not explain why high-level GUS activity using line LGS was dependent on the use of the transgenic line in the lower layer. This strong reliance of GUS activity in the fruiting body on transgenic mycelium in the lower layer combined with the absence of the GUS gene in the fruiting body tissue suggested a GUS element, either the protein or the RNA transcript, was translocated from the compost-born mycelium into the developing fruiting body.

### The translocated element is GUS protein and not RNA transcript

**Figure 3:**
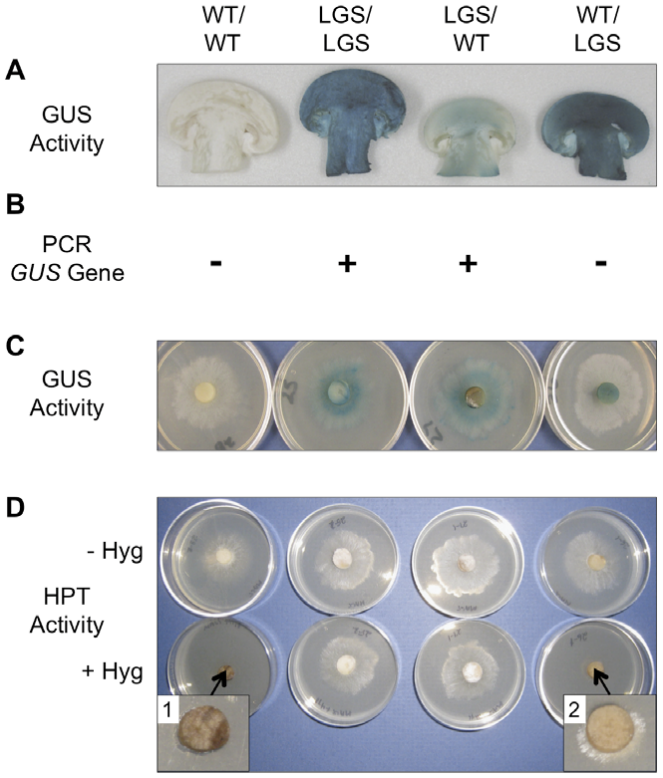
GUS and HPT activity assay of mycelial cultures derived from fruiting body tissue. Indicated is the upper layer inoculant/lower layer inoculant for the bi-layered cultivation substrate. WT: wild-type line; LGS: GUS line carrying the laccase 2 promoter. (A) Histological GUS assay and (B) *GUS*-PCR analysis of fruiting bodies. The presence (+) or absence (-) of the *GUS* gene is indicated. Mycelial tissue cultures derived from the indicated fruiting bodies were analyzed for: (C) GUS activity by histological assay and (D) Hyrgromycin phosphotransferase (HPT) activity. Hygromycin B resistance was assessed by growth on malt extract agar (MEA) without (- Hyg) and with (+ Hyg) 50 mg l^−1^ hygromycin B. Inserts 1 and 2: higher magnification of the fruiting body tissue discs indicated by the arrows.

While the genotype of the inoculant in the upper layer strictly governed the presence or absence of the *GUS* gene and RNA transcript in the fruiting body, this genotype was not highly predictive of GUS enzyme activity. Most notably, the WT/LGS and WT/AGS combinations produced fruiting bodies showing high GUS activity, but completely lacking the *GUS* transgene and transcript (Fig. 2E, F, G, H, I, J, K, L). The WT makeup of the GUS-expressing WT/LGS-fruiting body was confirmed by the absence of stably inherited GUS and hygromycin phosphotransferase (HPT) selectable marker activities in its derived mycelial tissue cultures (Fig. 3C, D) and basidiospores (Fig. S1). However, these mycelial cultures invariably displayed a passive HPT activity, suggesting translocation of an HPT element as well (Fig. 3D insert 2). Conversely, and as predicted by our hypothesis, stably inherited GUS and HPT activities were observed in mycelial cultures and basidiospores of LGS/LGS- and LGS/WT-fruiting bodies that were PCR-positive for the *GUS* gene.

The incongruity between the GUS genotype and phenotype can only be explained if the GUS enzyme was not synthesized in the fruiting body and upper-layer mycelium, but was transported as either protein or mRNA from the lower-layer mycelium. However, RT-PCR analysis showed that fruiting bodies with high-level GUS activity did not contain the *GUS* transcript; therefore, the translocated element must be the GUS protein. Based on this evidence, we hypothesized that organogenesis of the fruiting body in *A. bisporus* is associated with a process that enabled the movement of the GUS protein from the vegetative mycelium colonizing the lower compost layer into the developing reproductive tissues.

### Fruiting body phenotype is determined by the spatial position of the transgenic line

To more fully explore the long-distance movement of GUS protein, GUS expression patterns in the fruiting body were determined using transgenic lines HGS, LGS, and AGS as inoculants. Consistent with the observations from the studies described above supporting the translocation of GUS protein from the lower layer-mycelium to the fruiting body, fruiting bodies for the HGS/WT treatment retained ∼70% of the activity and those for the LGS/WT treatment<5% of the activity relative to their respective fully transgenic substrate treatment (Fig. 2A, B, E, F). These findings substantiated our hypothesis that the GUS protein passed from the LGS mycelium in the lower layer to the fruiting body. Similar to line HGS, line AGS showed an approximate 50% yield of GUS activity in fruiting bodies in treatment AGS/WT compared to AGS/AGS (Fig. 2I, J).

Each dual-inoculant treatment was also examined in the inverse conformation, placing the WT inoculant in the upper layer and GUS inoculant in the lower layer. This resulted in a reversal of the GUS expression pattern for the lines HGS and LGS. Fruiting bodies from the WT/HGS now retained<5% of the GUS activity (Fig. 2A, B), suggesting maximal enzyme activity was dependent on the mycelium of this line being directly involved in the formation of the fruiting body from the upper layer. On the other hand, WT/LGS-fruiting bodies yielded 80% of the maximal GUS activity (Fig. 2E, F), which was consistent with the translocation of the GUS protein from the compost-borne LGS mycelium. Line AGS proved largely unaffected by the spatial transposition of the inoculants, as fruiting bodies from AGS/WT and WT/AGS showed similar levels of GUS activity (Fig. 2I, J), which would be expected for a transgenic contributing equally from both layers. While the results of these studies further supported the long-distance movement of GUS protein, they also illustrated how differences in the GUS transgene promoter led to marked differences in the behavior of the transgenic inoculants.

### Fruiting body phenotype is determined by the tissue-specificity of the *GUS* gene promoter

Variation in GUS accumulation in fruiting bodies for different transgenic lines of *A. bisporus* can be explained by differences in the tissue-specificity of their promoters driving expression of the *GUS* gene. To help clarify this phenomenon, the tissue-preferred expression of the promoters used in this study was confirmed by a GUS activity assay of the mycelium colonizing the compost (Fig. S2), and was corroborated by a determination of the relative abundance of their cognate RNA transcripts based on a comparative transcriptome analysis (Table 1) and fruiting body cDNA library (Fig. S3). For example, the *LCC2* gene is highly expressed in the vegetative mycelium of *A. bisporus* [8], [9]. Consequently, in the WT/LGS treatment (Fig. 2E, F), line LGS carrying the strong mycelium-active *LCC2* promoter supported synthesis of the GUS protein in the lower layer (Fig. S2), which was then translocated to the fruiting body that developed exclusively from the WT mycelium in the upper layer. Inverting the inoculants (LGS/WT) produced fruiting bodies that contained the *GUS* gene, because the upper inoculant determined the genotype, but showed a markedly diminished GUS activity, because GUS protein was no longer translocated from the WT mycelium in the lower layer (Fig. 2E, F, G, H). The low GUS activity displayed by the latter fruiting bodies reflected a low-level expression in the fruiting body tissue and/or mycelium colonizing the thin upper layer of peat.

In contrast to the *LCC2* promoter, the *HYPA* promoter is highly active in the fruiting body, rather than the vegetative mycelium (Table 1; Figs. S2 and S3) [7]. As such, appreciable GUS activity only resulted when an HGS line was applied to the upper layer, where the fruiting body formed exclusively from its mycelium (Fig. 2A, B). The low-level GUS activity associated with fruiting bodies grown from WT/HGS inoculant combination reflected the translocation of a low-level of GUS that had been synthesized in the compost-borne mycelium (Fig. S2; Note: trace GUS activity was observed on the filter paper discs, but it is difficult to resolve in the photographic reproduction).

Finally, the combined data from the transcriptome analysis (Table 1), GUS activity assay of compost-borne mycelium (Fig. S2), and cDNA library (Fig. S3) indicated the constitutive expression of the *ACTN* gene in both the vegetative mycelium and reproductive fruiting body tissues in *A. bisporus*, which agrees with its behavior in other fungal species [10], [11]. Hence, constitutive expression explained why the AGS line produced appreciable GUS activity when paired with a WT inoculant positioned in either the upper or lower layer. GUS protein was synthesized and translocated from the lower-layer mycelium as well as synthesized *in situ* in the fruiting body and in the upper-layer mycelium (Fig. 2I, J).

### Protein translocation enables increased recombinant expression

**Figure 4:**
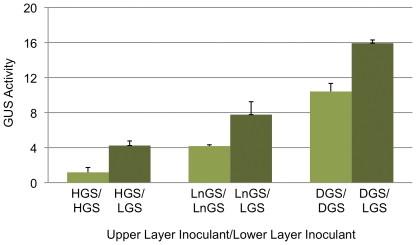
Use of protein translocation for increased recombinant protein production. Shown is the GUS activity in fruiting bodies grown using single-transgenic inoculant and dual-transgenic inoculant strategies. Indicated is the upper layer inoculant/lower layer inoculant for the bi-layered cultivation substrate. The single-inoculant treatment employed a line carrying a fruiting body-active promoter, either HGS (hydrophobin A promoter), LnGS (lectin promoter), or DGS (fruiting body-specific D promoter), as the inoculant for both layers. For the dual-inoculant treatment, the aforementioned lines were used as the upper-layer inoculants and individually paired with line LGS carrying the strong vegetative mycelium promoter, laccase 2, as the inoculant for the lower layer. GUS activity is expressed as nmol MUG hydrolyzed/minute/100 µg total soluble protein, and represents the mean value of two independent experiments. GUS activity for inoculant treatment WT/LGS = 3.77.

We explored the feasibility of achieving high-level GUS expression by simultaneous translocation of protein from the lower layer combined with *in situ* protein synthesis in the fruiting body. To this end, we independently paired an LGS line carrying the mycelium-preferred *LCC2* promoter as the lower-layer inoculant with three GUS lines carrying fruiting body-preferred promoters as the upper-layer inoculants. The latter lines were HGS, DGS carrying the fruiting body-specific D (*FBSD*) promoter, and LnGS carrying the lectin (*LCTN*) promoter (Table 1). Figure 4 demonstrates increased GUS activity resulted for each inoculant pair relative to the level obtained with the single inoculant. The increase in enzyme activity ranged from 1.5-fold for line DGS to 3.6-fold for line HGS. Thus, an appreciation for the protein translocation phenomenon enables the rationale design of transgene constructs for the purpose of achieving increased recombinant expression in *A. bisporus*.

### Protein translocation offers the potential for enhanced biocontainment of transgenic mushrooms

The ability to regulate the phenotype of the fruiting body independent of its genotype creates the unique opportunity to apply biotechnology for crop improvement. A transgenic line could be employed in the lower layer in the dual-inoculant strategy to translocate a protein conferring a desirable trait to an otherwise WT-fruiting body originating from the upper layer. In addition to the transgene-free status of the edible fruiting body (Figs. 2 and 3), this strategy would offer the advantage of enhanced biocontainment, as air-dispersed basidiospores would also be free of the transgene (Fig. S1).

### Long-distance protein translocation is a newly described phenomenon in *Agaricus bisporus*

To our knowledge, long-distance intercellular translocation of protein has not been described in filamentous fungi. Fungi carry out extracellular digestion of complex polymers in the environmental substrate through the action of an array of secreted hydrolytic enzymes, which is followed by the uptake and intercellular transport of simple molecules and low-molecular weight breakdown products to the actively growing hyphal tips in the mycelial network [12]. However, the recruitment of functional protein from the vegetative mycelial network in the compost substrate might offer the fungus a more favorable conservation of metabolic resources and increased speed of development relative to the complete reliance on the *de novo* synthesis of protein within the fruiting body. The translocation phenomenon may be analogous in principle to the conservative processes of autophagy [13], [14] and autolysis [15], [16], which are inherent in filamentous fungi and involve the reallocation of nutrients from mature mycelium to support nascent cell growth and development.

Our ability to so clearly observe intercellular protein translocation in *A. bisporus* was attributed to the unique bi-layered cultivation substrate and unexpected absence of extensive intermixing of WT and transgenic mycelia following their physical union at the interface of the two substrate layers. Cellular fusion of the two mycelial networks could conceivably signal apical-dominant polarized growth [17], [18] of the actively growing hyphal tips in the upper peat layer, which would account for the dominance of the upper-layer inoculant in determining the genotype of the fruiting body.

A remaining fundamental question is whether the directed mobilization of protein involves an active or passive source-sink process. For example, proteins might be transported by mass flow from the mycelium along a mannitol concentration gradient, which has been implicated in the osmotic influx of water into the developing fruiting body [19]–[21]. Alternatively, translocation could involve the reiteration of well-characterized intracellular processes for transport via the cytoskeleton [22] and tubular vesicles [23]. An intriguing possibility that warrants investigation is the rapid movement through specialized transport pathways, such as cords and rhizomorphs [20], [24]. Regardless of the precise process, translocation of the *GUS* protein, presumably in the 275-kDa homotetrameric active form, likely occurs via the dolipore septum, which is a perforation in the cross wall connecting adjacent hyphal cells [25].

### Conclusions

We have demonstrated the long-distance movement of a reporter protein from the vegetative mycelium in the compost into the developing fruiting body in *A. bisporus*, a finding that strongly suggests the transport of native proteins is also possible. Our *GUS* gene constructs did not contain a secretion signal (Fig. S4), which presumably localized the GUS protein to the cytosol. We are keenly interested in determining whether the translocation process is limited to cytosolic proteins or extends to ER-retained and secreted forms. Studies underway are focused on protein translocation as a means of affecting high-level expression of other recombinant proteins and conferring resistance to viruses associated with pathologies of cultivated *A. bisporus* [26] as well as exploring the transport of native proteins.

## Materials and Methods

### Fungal strains and growth media

Commercial intermediate-white hybrid strains of *A. bisporus* were used in this study. WT cultures were maintained on malt extract agar (MEA; 20 g l^−1^ malt extract, 2.1 g l^−1^ MOPS pH 7.0, 15 g l^−1^ agar) and transgenic cultures on MEA containing 50 mg l^−1^ hygromycin B (Sigma).

### Transgene constructs

*GUS* gene expression constructs were assembled in the *A. bisporus* transformation plasmid vector pBHg, which contained the *HPT* gene conferring resistance to hygromycin B as a selection marker [27]. Table 1. summarizes the various transgenic lines employed in the present study, including the sources and tissue preferences of the promoters used to drive expression of the *GUS* gene. Figure S4 depicts the general structure of the gene constructs and provides details on the gene elements.

### Fungal transformation

*Agrobacterium*-mediated transformation of *A. bisporus* lamellar tissue was carried out using bacterial strain AGL-1 and 30 mg l^−1^ hygromycin B for selection after the method of Chen, et al. [27] and as elaborated by Romaine and Schlagnhaufer [28].

### Preparation of mycelial inoculants

A 250-ml flask containing either 50 ml rye grain, 0.8 g calcium carbonate, 0.8 g calcium sulfate, and 60 ml Milli-Q water (lower layer inoculant) or 50 g proprietary matrix (Lambert Spawn Co., Coatesville, PA and Sylvan Inc., Kittanning. PA) and 75 ml Milli-Q water (upper layer inoculant) was autoclaved for 30 minutes. Each flask of inoculant was seeded with three mycelial agar blocks of *A. bisporus*, and maintained at room temperature for 2–3 weeks with occasional shaking to redistribute the inoculum.

### Fruiting body production

Fruiting bodies were cultivated as described elsewhere [6] except 15 g lower layer inoculant and 1.8 kg compost were mixed and then packed in a 25-cm-diameter plastic container, and 25 g upper layer inoculant and 1.5 l peat-based substrate were mixed and overlaid on the compost. Fruiting bodies were harvested, rinsed with water, diced, and stored as an aggregate sample by treatment at −20°C for PCR analysis and GUS assay and −78°C for RT-PCR analysis.

### Histological GUS assay

Longitudinal slices (2–3 mm thick) of freshly harvested fruiting bodies were incubated for 1.5–4 hrs at RT in 10 ml X-Gluc substrate (100 mM potassium phosphate pH 7.0, 25 mM ascorbic acid, 0.02% Triton X-100, 0.08% ethanol, 500 mg l^−1^ 5-bromo-4-chloro-indoxyl-β-D glucuronic acid (Gold Biotechnology, St. Louis, MO).

To analyze GUS enzyme activity in vegetative mycelium grown in compost, 1.75-cm holes were drilled into the sides of the 12-cm-diameter plastic pots containing compost used for fruiting body production, and moistened 2.3-cm-diameter Whatman 3MM filter paper discs were affixed with tape to cover the holes. After the two-week compost *Agaricus* colonization period, the discs were removed and placed in X-Gluc substrate for 2 hrs at RT.

To assess GUS activity in vegetative mycelium grown in axenic culture, a mycelial colony growing on MEA contained in a 6-cm-diameter Petri plate was flooded with 5 ml X-Gluc substrate and incubated overnight at RT.

### Quantitiative GUS assay

Frozen fruiting body tissue (3 g) was homogenized in 9 ml of extraction buffer (50 mM sodium phosphate pH 7.0, 10 mM EDTA, 10 mM mercaptoethanol, 0.1% Triton X-100, 0.1% sarkosyl) for 1 minute with a PT 10–35 GT polytron (Kinematica, Lucerne, Switzerland). The extract was clarified at 11,000 x *g* for 15 minutes and the protein concentration determined after the method of Bradford [29].

GUS activity was quantified by a fluorometric assay with a 4-methylumbeliferyl B–D-glucuronide (MUG; Sigma) substrate [30]. The value was reported as the mean of the ratio of the molar rate of formation of 4-methyl-7-hexacoumarin (MU) to the total soluble protein.

### HPT activity assay

To screen basidiospores for the co-transformed *HPT* gene conferring hygromycin B-resistant selection, a fruiting body approaching full maturity was soaked in a 10% commercial bleach solution (final concentration 0.6% NaClO) for 1 minute, and then rinsed exhaustively with sterile Milli-Q water. Using a scalpel, the stipe and velum were excised to expose the lamellae, and the pileus was suspended from a hooked wire over a sterilized 9-cm diameter filter paper disc within a sterilized glass chamber. After an overnight incubation at RT, the discharged basidiospores were washed from the surface of the paper with sterile Milli-Q water. A 100 µl aliquot of a turbid basidiospore suspension (>100,000 basidiospores/ml as determined by hemocytometry) was spread onto each of a 10-cm diameter Petri plate of MEA and MEA containing 100 mg l^−1^ hygromycin B, and the plates were incubated at room temperature for 3–4 weeks and observed for mycelial growth.

To assay fruiting body tissue for HPT activity, a 0.5-cm disc of internal pileus tissue was transferred aseptically onto each of a 6-cm-diameter Petri plate of MEA and MEA containing 50 mg l^−1^ hygromycin B. Plates were incubated at room temperature for 2–3 weeks at RT and observed for mycelial growth.

### Isolation of DNA

DNA was extracted from frozen fruiting body tissue (100 mg) using the LETS procedure [31] with a FastPrep FP-120 system (Thermo Fisher Scientific). DNA was stored in TE buffer (10 mM Tris-HCl, 2 mM EDTA, pH 8.0) at −78°C.

### Isolation of RNA

RNA was extracted from frozen fruiting body tissue (100 mg) using the RNAqueous Kit (Applied Biosystems) and FastPrep system. RNA was treated with 2 U DNAse in Tris-HCl pH 7.5, 2.5 mM MgCl_2_, 0.5 mM CaCl_2_ at 37°C for 1 hr, followed by standard phenol extraction and ethanol precipitation. RNA was stored in TE buffer at −78°C.

### PCR

Amplification was carried out in a final volume of 25 µl containing 0.75 U *Taq* DNA polymerase with Standard *Taq* Buffer (New England Biolabs), 200 µM dNTPs, 0.2 µM each primer and 10–50 ng DNA template. Primer set: Fwd 5′CGTGACAAGAACCATCC AAGCG3′ and Rev 5′GGGTAGCCATCACAAACAGCAC3′ were used to amplify a 163-bp sequence in the *GUS* gene. As a DNA template control, a separate reaction was run using primer set: Fwd 5′CGACGGGTGTGAACGCAAAGG3′ and Rev 5′CAATCAGTCG ATCAACGTTCGC3′, which defined a 403-bp sequence in the native polyphenol oxidase 1 (*PPO*) gene [32]. Thermocycling parameters were: 94°C for 5 minutes; 35 cycles of 94°C for 1 minute, 58°C for 1 minute and 72°C for 1 minute.

### RT-PCR

RT-PCR was performed (25 µl final volume) with a MasterAmp RT-PCR Kit (Epicentre Biotechnologies) using 30–100 ng RNA template and the PCR primer sets for the *GUS* and *PPO* genes in separate reactions. Thermocycling parameters were: 60°C for 20 minutes (RT); 94°C for 2 minutes; 40 cycles of 94°C for 1 minute and 60°C for 1 minute; 72°C for 7 minutes.

### qPCR

Real-time qPCR was carried out employing the standard protocol at the Huck Institute Genomics Core Facility, Penn State. *GUS* primers were: Fwd 5′CGACGGACTGACC ATCGAT3′ and Rev 5′GAACTTGCCGTCGTTGACTTC3′, with a 5′FAM-CCGTTCGGC GTGCGGACC3′BHQ probe sequence. *ACTN* primers were: Fwd 5′ATGCTCCTCGTG CCGTCTT3′ and Rev 5′TGCCCCATACCAACCATCA3′, with a 5′FAM-CCTTCCATCGT CGGTCGTCCTCG3′BHQ probe sequence. Amplification parameters were: 95°C for 10 minutes; 40 cycles of 95°C for 15 seconds and 60°C for 1 minute, using a 7300 Real-time Sequence Detection System (Applied Biosystems). Ct values of the *GUS* gene and the reference *ACTN* gene were used with the delta delta Ct method to determine relative levels.

### Fruiting body cDNA library

Total RNA was extracted from freshly harvested fruiting bodies using Tri-Reagent solution (Applied Biosystems/Ambion), and poly(A)^+^ RNA was isolated using oligo-(dT)_15_ cellulose (Promega). First-strand cDNAs were synthesized by reverse transcription using an oligo-dT primer with a *Xho I* restriction endonuclease site, which was followed by second-strand synthesis using DNA polymerase I in the presence of RNase H. Double-stranded cDNAs were ligated at their 5′-ends to an *EcoRI* adaptor and cloned in pBluescriptII XR vector (Agilent Technologies). A total of 4,608 bacterial colonies were spotted on Hybond-N membrane (GE Healthcare) and probed with synthesized gene-specific DNA oligos (60 nt) (Integrated DNA Technologies) that were labeled using an ECL Direct DNA/RNA Labeling/Detection Kit (GE Healthcare).

### Transcriptome analysis

A comparative transcriptomic analysis of fruiting body tissue and vegetative mycelium grown in compost for *A. bisporus* was conducted by Kerry Burton and associates of HRI, Warwick, UK (personal communication). Data were developed using an Agilent array of four replicates for each of fruiting body tissue and vegetative mycelium.

## Supporting Information

Figure S1 **HPT activity assay of basidiospores derived from fruiting bodies.** Indicated is the upper layer inoculant/lower layer inoculant for the bi-layered cultivation substrate. WT: wild-type line; LGS: transgenic GUS line carrying the laccase 2 promoter; HPT: hygromycin phosphotransferase. Basidiospores were collected from fruiting bodies grown using the indicated inoculant combinations and then plated on malt extract agar (MEA) without (- Hyg) and with (+ Hyg) 100 mg l^-1^ hygromycin B. Note: the LGS/LGS-fruiting body and WT/LGS-fruiting body were PCR-positive and PCR-negative, respectively, for the *GUS* gene.
(TIF)

Figure S2 **GUS enzyme activity in mycelium colonizing the lower compost layer.** For each treatment, three filter paper discs were exposed to the compost substrate during the *Agaricus bisporus* colonization period in the mushroom cultivation cycle and then subjected to a histological GUS assay. Compost inoculant treatments were: non-inoculated (None); WT-inoculated (WT) and inoculated with GUS lines carrying the hydrophobin A (HGS), laccase 2 (LGS), and β-actin (AGS) promoters.
(TIF)

Figure S3 **Colony blots of an** ***Agaricus bisporus*** **fruiting body cDNA library**. A cDNA library consisting of a total of 4,608 cDNA clones was screened by colony blot hybridization using gene-specific DNA oligos (60 nt) as probes. Based on probing∼4,800 bacterial colonies, the frequencies of cDNA sequences were: >110 for hydrophobin A *(HYPA)*,>50 for fruiting body-specific D (*FBSD)*; ∼20 for β-actin (*ACTN)*;<10 each for laccase 2 (*LCC2*) and lectin (*LCTN*).
(TIF)

Figure S4 **Structural organization of the GUS expression cassette.** The *HPT* gene, which conferred hygromycin B resistance as a selectable marker, and *GUS* reporter gene (*GUSPlus;* CambiaLabs) [33] were situated between the left (T-DNA LB) and right (T-DNA RB) border sequences of the *Agrobacterium* T-DNA. The *GUS* gene was joined to either the native hydrophobin A *(HYPA*), laccase 2 (*LCC2*), β-actin (*ACTN*), lectin (*LCTN*) or fruiting body-specific D (*FBSD*) promoter *(Promoter)*. *GUS* constructs contained the *HYPA* terminator sequence (*Terminator*), except for the *FBSD* construct, which incorporated the *Arabidopsis* polyubiquitin gene terminator [34]. The positions of the *HYPA* introns within the *GUS* gene are shown. The *HPT* gene was linked to the native glyceraldehyde 3-phosphate dehydrogenase promoter (*GPD-P*) and *Cauliflower mosaic virus* 35S terminator (*35S-T*) [27], [28]. Promoter sequences of 552 bp and 1270 bp were isolated from the native *HYPA* [7] and *LCC2* [9] genes, respectively, by PCR amplification of the DNA sequence directly upstream of the respective start codon. 2-D profiling of fruiting body proteins identified LCTN and FBSD, and their cDNA sequences identified by a blast search using peptide sequences. The *ACTN* cDNA sequence was identified from published data. DNA fragments of 578 bp, 701 bp, and 804 bp, situated directly upstream of the respective start codon, were obtained by PCR-based genome walking and employed as promoters for the *LCTN*, *FBSD* and *ACTN* genes, respectively. *GUSPlus*, which was modified by the addition of *HYPA* introns 2 and 3, was linked to a native promoter sequence at the *Nco I* restriction site. PCR-amplified sequences of 183 bp and 405 bp, downstream of the respective stop codon for the *HYPA* gene and *Arabidopsis* polyubiquitin gene, respectively, were used as terminators.
(TIF)
