# Supplementary figures and images for: Long-Distance Translocation of Protein during Morphogenesis of the Fruiting Body in the Filamentous Fungus, Agaricus bisporus
Source: PLoS One. 2011 Dec 6;6(12):e28412. doi: 10.1371/journal.pone.0028412 (PMC3232199; doi:10.1371/journal.pone.0028412)

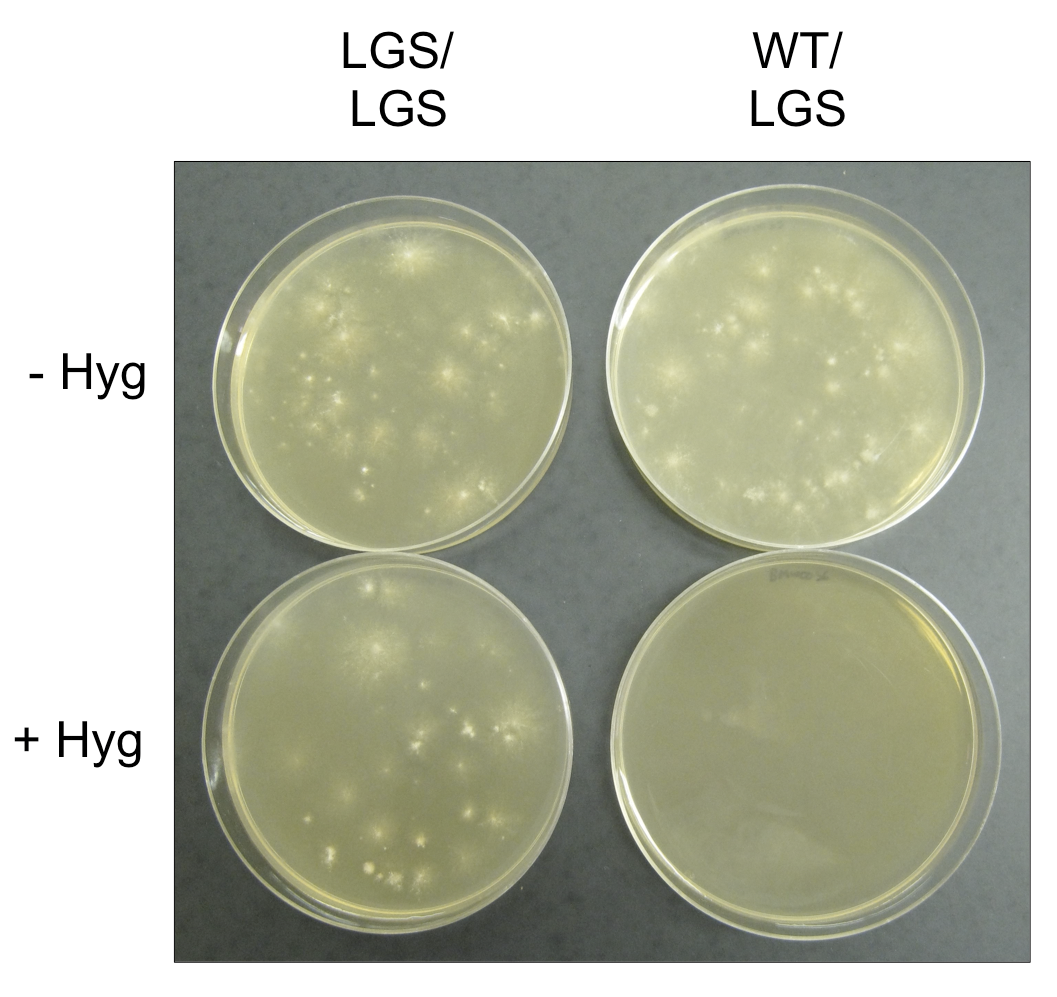

Supplement: Figure S1 — HPT activity assay of basidiospores derived from fruiting bodies. Indicated is the upper layer inoculant/lower layer inoculant for the bi-layered cultivation substrate. WT: wild-type line; LGS: transgenic GUS line carrying the laccase 2 promoter; HPT: hygromycin phosphotransferase. Basidiospores were collected from fruiting bodies grown using the indicated inoculant combinations and then plated on malt extract agar (MEA) without (- Hyg) and with (+ Hyg) 100 mg l-1 hygromycin B. Note: the LGS/LGS-fruiting body and WT/LGS-fruiting body were PCR-positive and PCR-negative, respectively, for the GUS gene. (TIF) [file pone.0028412.s001.tif]

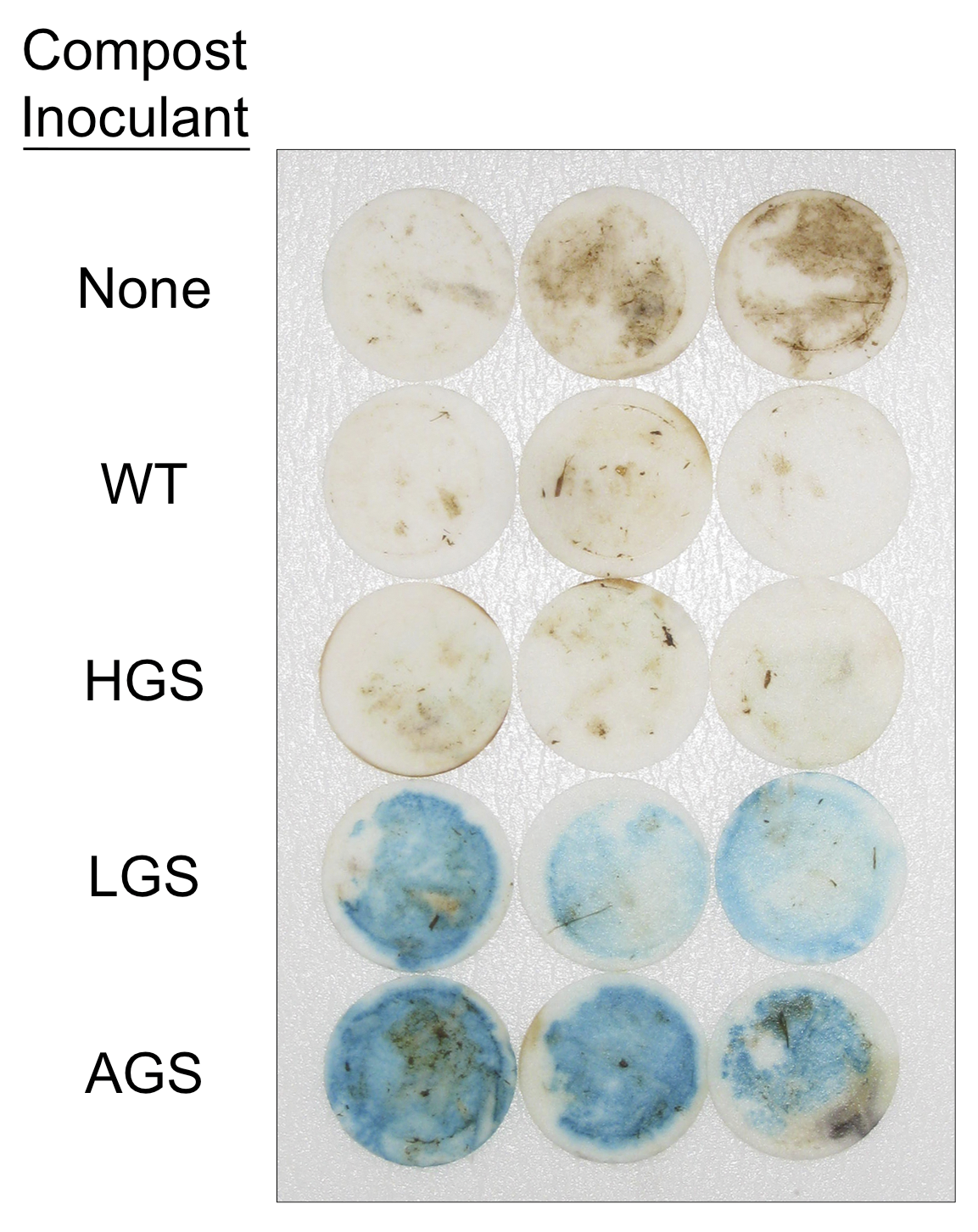

Supplement: Figure S2 — GUS enzyme activity in mycelium colonizing the lower compost layer. For each treatment, three filter paper discs were exposed to the compost substrate during the Agaricus bisporus colonization period in the mushroom cultivation cycle and then subjected to a histological GUS assay. Compost inoculant treatments were: non-inoculated (None); WT-inoculated (WT) and inoculated with GUS lines carrying the hydrophobin A (HGS), laccase 2 (LGS), and β-actin (AGS) promoters. (TIF) [file pone.0028412.s002.tif]

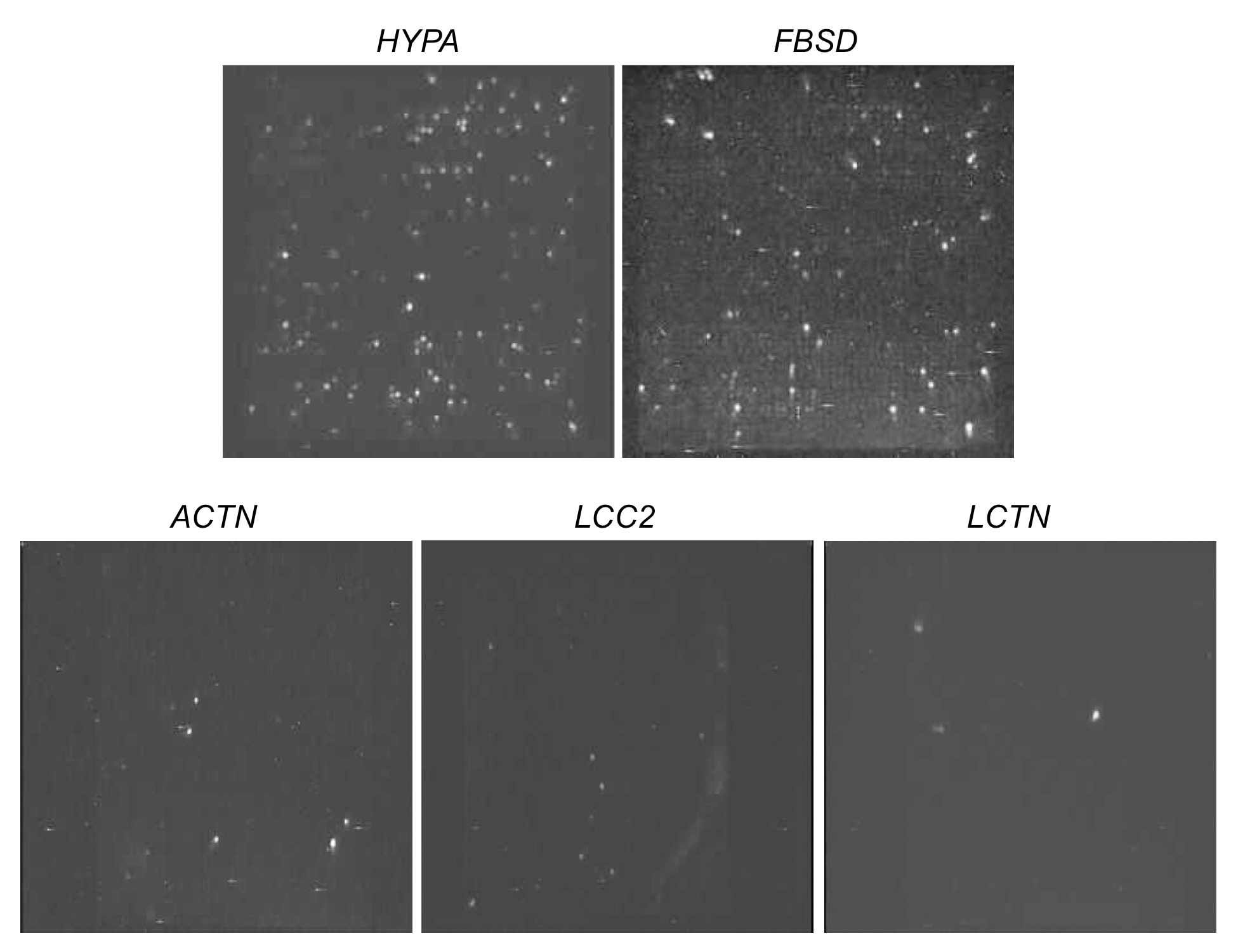

Supplement: Figure S3 — Colony blots of an Agaricus bisporus fruiting body cDNA library. A cDNA library consisting of a total of 4,608 cDNA clones was screened by colony blot hybridization using gene-specific DNA oligos (60 nt) as probes. Based on probing∼4,800 bacterial colonies, the frequencies of cDNA sequences were: >110 for hydrophobin A (HYPA),>50 for fruiting body-specific D (FBSD); ∼20 for β-actin (ACTN);<10 each for laccase 2 (LCC2) and lectin (LCTN). (TIF) [file pone.0028412.s003.tif]

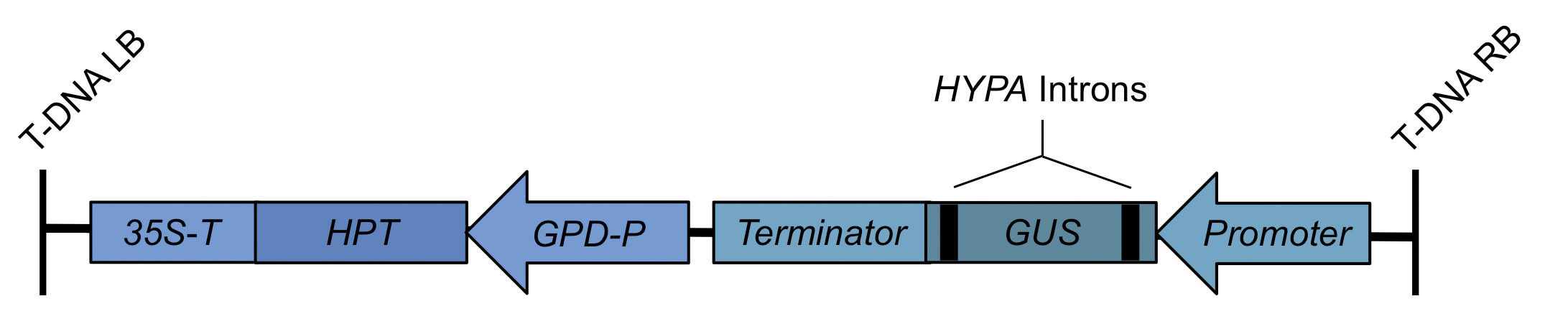

Supplement: Figure S4 — Structural organization of the GUS expression cassette. The HPT gene, which conferred hygromycin B resistance as a selectable marker, and GUS reporter gene (GUSPlus; CambiaLabs) [33] were situated between the left (T-DNA LB) and right (T-DNA RB) border sequences of the Agrobacterium T-DNA. The GUS gene was joined to either the native hydrophobin A (HYPA), laccase 2 (LCC2), β-actin (ACTN), lectin (LCTN) or fruiting body-specific D (FBSD) promoter (Promoter). GUS constructs contained the HYPA terminator sequence (Terminator), except for the FBSD construct, which incorporated the Arabidopsis polyubiquitin gene terminator [34]. The positions of the HYPA introns within the GUS gene are shown. The HPT gene was linked to the native glyceraldehyde 3-phosphate dehydrogenase promoter (GPD-P) and Cauliflower mosaic virus 35S terminator (35S-T) [27], [28]. Promoter sequences of 552 bp and 1270 bp were isolated from the native HYPA [7] and LCC2 [9] genes, respectively, by PCR amplification of the DNA sequence directly upstream of the respective start codon. 2-D profiling of fruiting body proteins identified LCTN and FBSD, and their cDNA sequences identified by a blast search using peptide sequences. The ACTN cDNA sequence was identified from published data. DNA fragments of 578 bp, 701 bp, and 804 bp, situated directly upstream of the respective start codon, were obtained by PCR-based genome walking and employed as promoters for the LCTN, FBSD and ACTN genes, respectively. GUSPlus, which was modified by the addition of HYPA introns 2 and 3, was linked to a native promoter sequence at the Nco I restriction site. PCR-amplified sequences of 183 bp and 405 bp, downstream of the respective stop codon for the HYPA gene and Arabidopsis polyubiquitin gene, respectively, were used as terminators. (TIF) [file pone.0028412.s004.tif]
